# Opportunities for optimizing fungal biological control agents for long-term and effective management of insect pests of orchards and vineyards: a review

**DOI:** 10.3389/ffunb.2024.1443343

**Published:** 2024-08-01

**Authors:** Christopher M. Wallis, Mark S. Sisterson

**Affiliations:** Crop Diseases, Pest and Genetics Research Unit, San Joaquin Valley Agricultural Sciences Center, U.S. Department of Agriculture – Agricultural Research Service, Parlier, CA, United States

**Keywords:** biological control, entomopathogenic fungi, *Beauveria* spp., *Metarhizium* spp., grapevine, citrus, *Prunus* spp.

## Abstract

Novel tactics for controlling insect pests in perennial fruit and nut crops are needed because target pests often display decreased susceptibility to chemical controls due to overreliance on a handful of active ingredients and regulatory issues. As an alternative to chemical controls, entomopathogenic fungi could be utilized as biological control agents to manage insect pest populations. However, development of field ready products is hampered by a lack of basic knowledge. Development of field ready products requires collecting, screening, and characterizing a greater variety of potential entomopathogenic fungal species and strains. Creation of a standardized research framework to study entomopathogenic fungi will aid in identifying the potential mechanisms of biological control activity that fungi could possess, including antibiotic metabolite production; strains and species best suited to survive in different climates and agroecosystems; and optimized combinations of entomopathogenic fungi and novel formulations. This mini review therefore discusses strategies to collect and characterize new entomopathogenic strains, test different potential mechanisms of biocontrol activity, examine ability of different species and strains to tolerate different climates, and lastly how to utilize this information to develop strains into products for growers.

## 1 Introduction

Acreage of perennial crops, including grapevines, fruit trees, and nut crops is increasing because perennial fruit and nut crops provide greater returns than field and forage crops. However, preventing pests and diseases from reducing yields in monocultures held for a decade or longer is challenging. Abundance of insect pests, and the pathogens they may transmit, may increase each season leading to a gradual decline in yields, eventually resulting in complete vineyard or orchard removal (Mustu et al., 2015). Most pest management programs rely on synthetic chemical controls. However, overreliance on a handful of active ingredients has resulted in decreased susceptibility in target pests, particularly when active ingredients are utilized repeatedly (Mustu et al., 2015; Sharma et al., 2018). In addition, regulatory agencies have called for the reduction or limitation of synthetic chemical-based pesticides due to environmental and human health concerns, and, coupled with the increased cost of developing new, safer synthetic pesticides (up to $250 million), there is an increasingly smaller variety of such products on the market (Glare et al., 2012). As a result, an increasing number of growers are turning to non-synthetic pesticides and other organic practices to serve a burgeoning market (Glare et al., 2012; Lacey et al., 2015). Therefore, there is a need to develop alternative methods to decrease pest insect populations that reduce fruit and nut crop yields.

One tactic to manage insect pests that has demonstrated some success is the use of biopesticides developed from entomopathogenic fungi (Lacey et al., 2001; Da Silva Santos et al., 2022; Irsad et al., 2023). Several entomopathogenic products are available on the market targeting a range of pests in perennial fruit and nut crops (Faria and Wraight, 2007; Da Silva Santos et al., 2022). Most currently available products involve one of four fungal genera: *Beauveria* (e.g. De La Rosa et al., 2000; Wraight et al., 2007a, 2007b; Keller et al., 2003; Brownbridge et al., 2006; Townsend et al., 2010), *Isaria* (e.g. Wraight et al., 2007b; Zimmermann, 2008; Lacey et al., 2011), *Akanthomyces* (formerly *Lecanicillium*) (e.g. Goettel et al., 2008; Kim et al., 2009), or *Metarhizium* (e.g. De La Rosa et al., 2000; Lomer et al., 2001; Chandler et al., 2005; Lacey et al., 2011; Jaronski and Jackson, 2012). However, formulations involving other species also have been developed such as *Aschersonia aleyrodis*, *Conidiobolus thromboides*, *Hirsutella thompsonii* and *Nomuraea rileyi* (Faria and Wraight, 2007; Lacey et al., 2015).

While commercial formulations for entomopathogenic fungi are available, including those using species in the genera *Beauveria* sp., *Isaria* sp., and *Metarhizium* sp., additional screening and testing is needed to identify novel virulent isolates and expand the overall diversity of described strains. Further research is warranted to clarify entomopathogenic fungi-host-microbiota interactions using modern molecular biology techniques such next-generation sequencing. Recent progress on understanding the role of environment on the effectiveness of entomopathogenic fungi, as review by Lacey et al. (2015), should continue as it will be needed to ensure overall effectiveness of products. Similarly, testing of mixtures of isolates is needed to identify synergistic effects as only a handful of studies have reported on research utilizing this approach (e.g. Spescha et al., 2023a, 2023b). Furthermore, once virulent isolates are identified, considerable testing is required to optimize formulations, application rates and methods using up-to-date research approaches. Here in this mini-review, the current state of entomopathogenic fungi research is examined, with the ultimate focus on improvement of the use of fungal biological control agents to limit abundance of insect pests in perennial crops.

## 2 Improving collection strategies to obtain a greater diversity of entomopathogenic fungi

Entomopathogenic fungi are typically collected from a single location at a single point in time. The procedure involves collecting the target pest, surface sterilizing bodies, and holding surface sterilized bodies on isolation medium (Da Silva Santos et al., 2022). This approach has been used to identify numerous entomopathogenic fungi that have been tested as pure strains, with studies targeting piercing-sucking insects (Brownbridge et al., 2001; Meekers et al., 2002; Cuthbertson and Walters, 2005; Nielsen and Hajek, 2005; Labbe et al., 2009; Lacey et al., 2011), chewing insects (Zimmermann, 1992; Lomer et al., 1999, 2001; De La Rosa et al., 2000; Thomas, 2000; Wraight and Ramos, 2002; Chandler and Davidson, 2005; Brownbridge et al., 2006; Dolci et al., 2006; Hajek, 2007; Moscardi and Sosa-Gomez, 2007; Townsend et al., 2010; Thakre et al., 2011), and other arthropods such as mites (Chandler et al., 2000, 2005; Wekesa et al., 2005; Abolins et al., 2007). Despite these efforts, most strains have been isolated from insect pests of non-woody host plants. However, there are a limited number of studies conducted on pests of woody plants such as those conducted by Hajek (2007) that targeted the spongy moth, *Lymantria dispar.* Further, many studies focus on optimizing use of entomopathogenic fungi to control pests in a contained environment such as a greenhouse, with research often on whiteflies (Aleyrodidae) and mites (Chandler et al., 2005; Labbe et al., 2009).

Recently, some entomopathogenic fungal strains have been isolated from insect pests of woody crops and tested for virulence. For instance, *Beauveria bassiana*, *Isaria fumosorosea*, *Metarhizium anisopliae* and/or *Metarhizium robertsii* strains have been identified that kill pests of grapevine including the European grapevine moth (*Lobesia botrana*) (Aguilera Sammaritano et al., 2018; Aguilera Sammaritano et al., 2021; Lopez Plantey et al., 2019; Beris et al., 2024), planthoppers (Moussa et al., 2021), vine mealybug (Rondot and Reineke, 2018), and grapevine aphid (Sayed et al., 2020). For orchard pests, *Beauveria bassiana*, *Isaria fumosorosea*, *Metarhizium anisopliae* and/or *Podonectria* sp. strains have been isolated from fruit flies (Goble et al., 2011), moths (Coombes et al., 2016), psyllids (Gandarilla-Pacheco et al., 2013), scale insects (Dao et al., 2016), and others (Shapiro-Ilan et al., 2003; Pereault et al., 2009).

**Figure 1:**
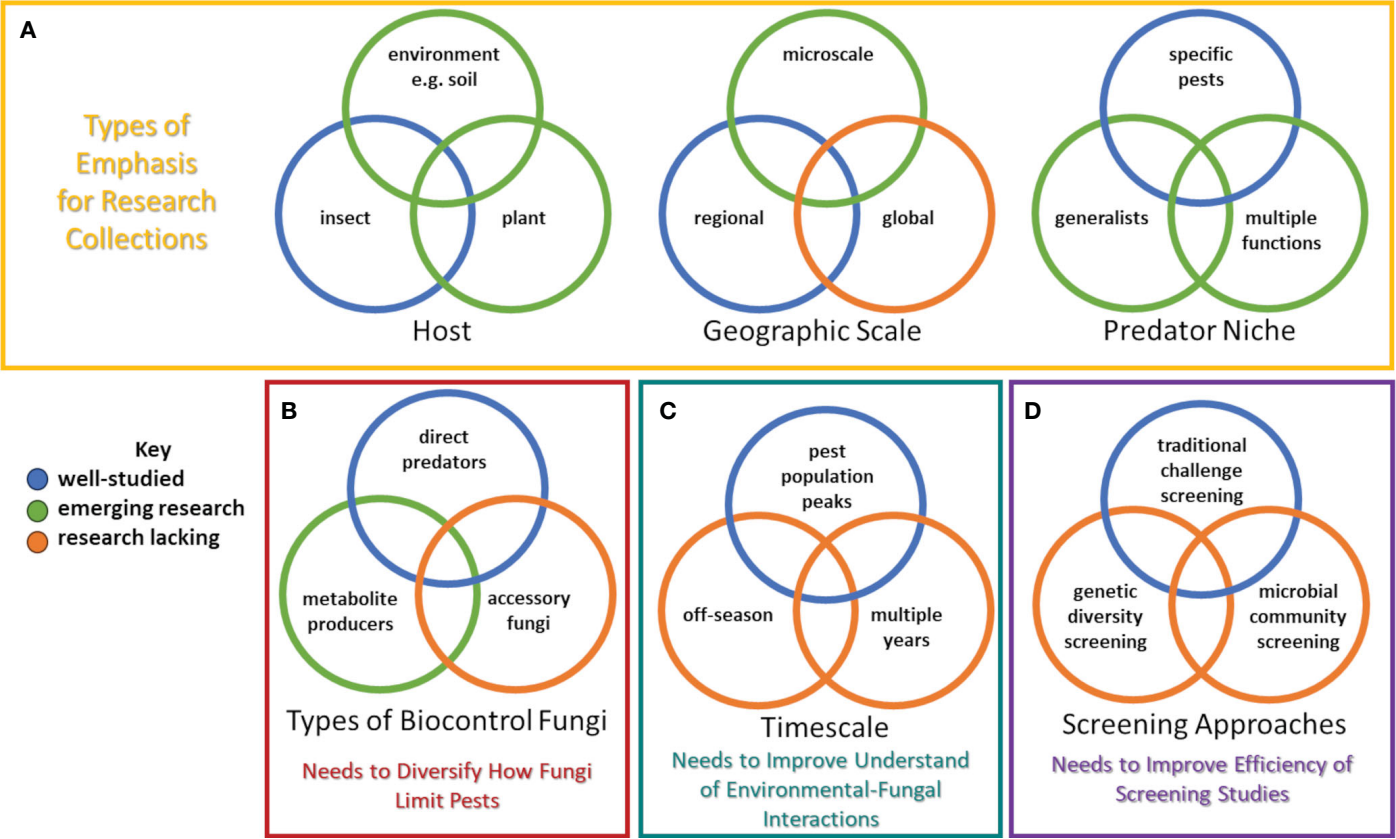
Research needed to robustly develop entomopathogenic fungi as biopesticides: **(A)** collection of new strains, **(B)** characterizing how certain fungal strains limit insect populations, **(C)** examining fungal-environmental interactions, and **(D)** screening of strains. Colors indicate areas that are well-researched, involve emerging interest, or where research is still lacking. Note that other research efforts beyond these are needed, and overlap exists between these types of research.

Despite recent progress, considerable advancement is needed to realize the full potential of using entomopathogenic fungi to control pests in perennial fruit and nut crops. A concerted effort to obtain and evaluate a greater number of entomopathogenic fungi from woody perennial plants would aid in developing a more diverse collection and associated data that could be used to improve understanding about entomopathogenic fungi in many ways (**Figure 1A**). While studies should continue to isolate fungi directly from target pests, additional sampling to isolate fungi directly from plant tissue or the environment should also be conducted. Indeed, recent attempts to discover strains that may manage vineyard and orchard pest populations have used isolates collected from plants or the environment (often soil) (Goble et al., 2011; Lopez Plantey et al., 2019). For the former, plant tissues could be pulverized after surface sterilization, and then mixed into sterile media (Ownley et al., 2008; Da Silva Santos et al., 2022). For the latter, soil could serially diluted and plated on selective media (containing specific compounds or antibiotics) (Luz et al., 2007; Rocha and Luz, 2009). Insect baits could also be used to acquire entomopathogenic fungi from soil (Goble et al., 2010; Vega et al., 2012; Lopez Plantey et al., 2019).

Isolates are typically collected from a single growing region and climate. However, strains that can be marketed to a wider consumer base, for instance in multiple regions and climates, are more likely to be viewed as economically viable because mass production of entomopathogenic fungi can be expensive (de Faria and Wraight, 2007; Jaronski and Mascarin, 2017; Marrone, 2019). Thus, it would be useful to identify strains that could be applied across wide geographic areas, and this requires collaboration across countries and continents (Kabaluk et al., 2010).

While identifying strains that can be applied across a large geographic scale is important, optimizing control requires understanding effects of microclimate on performance of entomopathogens (Marrone, 2014; Maina et al., 2018). Because sampling at the microenvironmental scale is key to understanding interactions between strains of entomopathogenic fungi and the environment, this topic will be discussed in more detail later in this review.

## 3 Entomopathogenic fungi-host-microbiota interactions

Often, entomopathogenic fungi that show promise in controlled laboratory settings fail to be successful in the field or nature settings (Vega et al., 2012). Poor performance of entomopathogenic fungi could be due to environmental effects (discussed later) or due to intricate insect-fungal interactions. To better understand fungal-insect interactions, studies have been conducted to identify how fungi colonize the host insect and mechanisms of insect resistance (Da Silva Santos et al., 2022). The mechanisms that entomopathogenic fungi utilized have been well reviewed (e.g. Inglis et al., 2001; Charnley, 2003; Charnley and Collins, 2007; Ortiz-Urquiza and Keyhani, 2013; Singh et al., 2017; Ma et al., 2024). Likewise, research on insect immune response to infection has been conducted (Qu and Wang, 2018; Da Silva Sanots et al., 2022). Mechanisms that entomopathogenic fungi use to overcome insect immune response include masking colonization (Wang and Leger, 2006), possessing resistance to antifungal compounds (Lu et al., 2015), production of enzymes that better penetrate insect cuticles and tissues (Ali et al., 2010), and production of secondary metabolites that weaken host immune responses (Pal et al., 2007; Xu et al., 2017). Additional screening of metabolites for inclusion in novel formulations of biopesticides is needed as such compounds could affect insect behavior, development, or survival.

In addition to studies on how entomopathogenic fungi may act as direct predators or produce toxic metabolites that kill insects, research on the interaction of entomopathogenic fungi with the microbial community in and around the insect is needed (**Figure 1B**). Wei et al. (2017) observed how *Beauveria bassiana* interacted with insect gut microbiota in mosquito hosts to ultimately result in death. Accordingly, research to identify and describe interactions among fungal community members found in vineyard and orchard settings is warranted. Studies should focus on determining whether microbial endophytes or epiphytes of insects interact with entomopathogenic fungi to have synergistic effects. Utilizing next-generation sequencing technologies to examine insect microbiomes could greatly increase understanding of accessory microbes that work with entomopathogenic fungi to infect hosts (Gurung et al., 2019; Gupta and Nair, 2020).

## 4 Research to clarify environmental adaptations of fungi

Key to commercially producing and deploying entomopathogenic fungi as biopesticides is understanding how entomopathogenic fungi survive and thrive in different environments. Several studies have examined effects of environmental conditions on entomopathogenic fungi such as effects of soil composition (Milner et al., 2003; Bruck, 2005; Quesada-Moraga et al., 2007; Roy et al., 2010a, 2010b), agricultural practices (Hummel et al., 2002; Townsend et al., 2003), and the capacity of entomopathogenic fungi to grow on or in the plants that the targeted pests feed upon (Inyang et al., 1998; Ownley et al., 2004, 2010; Ugine et al., 2007a, 2007b; Olleka et al., 2009; Cory and Ericsson, 2010). The ability of entomopathogenic fungi to colonize plants is of particular interest because it may provide the opportunity to kill target pests and prevent colonization of the plant by bacterial, fungal, nematode, or viral pathogens (Cherry et al., 2005; Ownley et al., 2004; Ownley et al., 2010; Brownbridge, 2006; Quesada-Moraga et al., 2009; Kim et al., 2009; Koike et al., 2011).

Despite a collection of research focused on understanding microclimatic effects on entomopathogenic fungi and the capacity of some fungal strains to adopt different lifestyles (i.e. as an endophyte in crop plants or ability to dwell in the soil as a saprophyte), little is known about the temporal dynamics on entomopathogenic fungal populations, especially whether they peak with targeted pest populations, what the fate of the entomopathogenic fungi is during the dormant season, and how populations may flux over multiple years (**Figure 1C**). Most applications of entomopathogenic fungi are made in response to observations of high pest abundance (Lacey et al., 2015). Yet, with perennial crops it would be advantageous to develop entomopathogenic fungi that could colonize the vineyard or orchard for multiple years, avoiding the need for re-applications and providing a baseline level of control (Meyling and Eilenberg, 2007; Pell, 2007). Some research has been conducted on approaches to conserve entomopathogenic fungi in the environment, thereby facilitating natural epizootics (Steinkraus, 2007a, 2007b; Pell et al., 2010). Sampling throughout the year for entomopathogenic fungi in different areas of the orchard could reveal where entomopathogenic fungi dwell when their insect hosts are not present (Lacey et al., 2015). Likewise, monitoring the dynamics of applied or natural entomopathogenic fungi over years in a vineyard or orchard environment may reveal which fungi are best suited for long-term, baseline control for insect pests (Lacey et al., 2015).

## 5 Improving entomopathogenic fungi-based product formulations

Selecting the best entomopathogenic fungi and determining the optimal formulation to make and disperse inoculum is key for their use as biopesticides (Santoro et al., 2005; Da Silva Santos et al., 2022). Selection generally involves the following: observing fungal virulence, quantifying reproductive capacity, assessing ability to mass produce, evaluating viability during storage and application, and rating effectiveness and survival after application (Ambethgar, 2009; Lopes et al., 2011).

Methods to perform the screening described above are well established (Da Silva Santos et al., 2022). However, the advent of modern genomic approaches and next-generation sequencing presents new opportunities to not only improve selections via traditional screenings but also by providing new tools to conduct novel experiments to advance our understanding of fungal genetic diversity and assessing entire microbial communities (**Figure 1D**). For instance, examination of effective and less-effective strains of the same or different entomopathogenic fungi species could identify genes and quantitative trait loci that are linked to improved virulence, reproduction, and survival in vineyards or orchards. Once these genes are discovered, newly collected strains could be quickly screened to observe if desired traits are present.

A combination of entomopathogenic fungi, or other non-fungal insect pathogens may be incorporated into products, providing synergistic effects (Malusa et al., 2021; Spescha et al., 2023a, 2023b). Knowledge about which microorganisms naturally co-occur in the environment is key to determining which microorganisms may need to be included together in a final product designed to have persistent, long-term control. Indeed, this has been attempted on several occasions with studies targeting soil pests (Bueno-Pallero et al., 2018; Spescha et al., 2023a), greenhouse pests (Shapiro-Ilan et al., 2004), and moths (Wang et al., 2021). Using next-generation sequencing of genomic DNA extracted from insect pests, crop plants, and the environment may reveal species that naturally co-occur, suggesting consideration for inclusion in a multiple-microorganism biopesticide formulation (Spescha et al., 2023b).

In addition to combinations of microorganisms, formulations of biopesticides could also contain biorational or other compounds, produced naturally by fungal isolates, to improve pest control. Such compounds could be identified via metabolomics studies of the different entomopathogenic fungi or associated fungi/microorganisms, and then added to the formulations for improved control (Berestetskiy and Hu, 2021). Accordingly, research should aim to isolate and identify metabolites from entomopathogenic fungi that possess insecticidal activity, via chromatography-based techniques such as those described by Elbanhawy et al. (2019) that analyzed methanolic extracts and quantified specific fatty acids. Follow-up research to then mass-produce and incorporate metabolites into biopesticide formulations would then need to occur.

## 6 Conclusions

Decreased effectiveness of overused insecticides and regulatory issues make controlling many insect pests in perennial crops challenging. Entomopathogenic fungal products provide an alternative strategy that could be integrated into management programs. Recent advances in genomics, proteomics, and metabolomics provide important tools that can be leveraged to identify useful strains and synergistic interactions.

## Author contributions

CW: Writing – original draft, Writing – review & editing. MS: Writing – review & editing.

## References

[B1] Abolins S. Thind B. Jackson V. Luke B. Moore D. Wall R.. (2007). Control of the sheep scab mite *Psoroptes ovis in vivo* and *in vitro* using fungal pathogens. Vet. Parasitol. 148, 310–317. doi: 10.1016/j.vetpar.2007.06.008 17624674

[B2] Aguilera-Sammaritano J. Caballero J. Deymié M. Rosa M. Vazquez F. Pappano D.. (2021). Dual effects of entomopathogenic fungi on control of the pest *Lobesia botrana* and the pathogenic fungus *Eutypella microtheca* on grapevine. Biol. Res. 54, 44. doi: 10.1186/s40659-021-00367-x 34952648 PMC8709985

[B3] Aguilera Sammaritano J. Deymie M. Herrera M. Vazquez F. Cuthbertson A. G. S. Lopez-Lastra C.. (2018). The entomopathogenic fungus, *Metarhizium anisopliae* for the European grapevine moth, *Lobesia botrana* Den. & Schiff. (Lepidoptera: Tortricidae) and its effect to the phytopathogenic fungus, *Botrytis cinerea* . Egypt J. Biol. Pest Control 28, 83. doi: 10.1186/s41938-018-0086-4

[B4] Ali S. Huang Z. Ren S. (2010). Production of cuticle degrading enzymes by *Isaria fumosorosea* and their evaluation as a biocontrol agent against diamondback moth. J. Pestic. Sci. 83, 361–370. doi: 10.1007/s10340-010-0305-6

[B5] Ambethgar V. (2009). Potential of entomopathogenic fungi in insecticide resistance management (IRM): a review. J. Biopest. 2, 177–193. doi: 10.57182/jbiopestic

[B6] Berestetskiy A. Hu Q. (2021). The chemical ecology approach to reveal fungal metabolites for arthropod pest management. Microorganisms 9, 1379. doi: 10.3390/microorganisms9071379 34202923 PMC8307166

[B7] Beris E. Papachristos D. Ponchon M. Caca D. Kontodimas D. Reineke A. (2024). The effects of temperature on pathogenicity of entomopathogenic fungi for controlling larval populations of the European grapevine moth (*Lobesia botrana*) (Lepidoptera: Torticidae). Crop Prot. 177, 106542. doi: 10.1016/j.cropro.2023.106542

[B8] Brownbridge M. (2006). Entomopathogenic fungi: status and considerations for their development and use in integrated pest management. Recent Res. Dev. Entomol. 5, 27–58.

[B9] Brownbridge M. Costa S. Jaronski S. T. (2001). Effects of *in vitro* passage of *Beauveria bassiana* on virulence to *Bemisia tabaci* . J. Invertebr. Pathol. 77, 280–283. doi: 10.1006/jipa.2001.5020 11437531

[B10] Brownbridge M. Nelson T. L. Hackell D. L. Eden T. M. Wilson D. J. Willoughby B. E.. (2006). Field application of biopolymer-coated *Beauveria bassiana* F418 for clover root weevil (*Sitona lepidus*) control in Waikato and Manawatu. N. Z. Plant Protect. 59, 304–311. doi: 10.30843/nzpp.2006.59.4481

[B11] Bruck D. J. (2005). Ecology of *Metarhizium anisopliae* in soilless potting media and the rhizosphere: implications for pest management. Biol. Control 32, 155–163. doi: 10.1016/j.biocontrol.2004.09.003

[B12] Bueno-Pallero F. A. Blanco-Pérez R. Dionisio L. Campos-Herrera R. (2018). Simultaneous exposure of nematophagous fungi, entomopathogenic nematodes and entomopathogenic fungi can modulate belowground insect pest control. J. Invertebr. Pathol. 154, 85–94. doi: 10.1016/j.jip.2018.04.004 29634923

[B13] Chandler D. Davidson G. (2005). Evaluation of entomopathogenic fungus *Metarhizium anisopliae* against soil-dwelling stages of cabbage maggot (Diptera: Anthomyiidae) in glasshouse and field experiments and effect of fungicides on fungal activity. J. Econ. Entomol. 98, 1856–1186. doi: 10.1093/jee/98.6.1856 16539104

[B14] Chandler D. Davidson G. Jacobson R. J. (2005). Laboratory and glasshouse evaluation of entomopathogenic fungi against the two-spotted spider mite, *Tetranychus urticae* (Acari: Tetranychidae), on tomato, *Lycopersicon esculentum* . Biocontrol Sci. Technol. 15, 37–54. doi: 10.1080/09583150410001720617

[B15] Chandler D. Davidson G. Pell J. K. Ball B. V. Shaw K. Sunderland K. D. (2000). Fungal biocontrol of acari. Biocontrol Sci. Technol. 10, 357–384. doi: 10.1080/09583150050114972

[B16] Charnley A. K. (2003). Fungal pathogens of insects: cuticle degrading enzymes and toxins. Adv. Bot. Res. 40, 241–321. doi: 10.1016/S0065-2296(05)40006-3

[B17] Charnley A. K. Collins S. A. (2007). ““Entomopathogenic fungi and their role in pest control,”,” in Environmental and Microbial Relationships: The Mycota IV, 2nd ed. Eds. Kubicek C. P. Druzhinina I. S. (Springer-Verlag, Berlin), 159–187.

[B18] Cherry A. J. Abalo P. Hell K. (2005). A laboratory assessment of the potential of different strains of the entomopathogenic fungi *Beuveria bassiana* (Balsamo) Vuillemin and *Metarhizium anisopliae* (Metschnikoff) to control *Callosobruchus maculatus* (F.) (Coleoptera: Bruchidae) in stored cowpea. J. Stored Prod. Res. 41, 295–309. doi: 10.1016/j.jspr.2004.04.002

[B19] Coombes C. A. Hill M. P. Moore S. D. Dames J. F. (2016). Entomopathogenic fungi as control agents of *Thaumatotibia leucotreta* in citrus orchards: field efficacy and persistence. BioControl 61, 729–739. doi: 10.1007/s10526-016-9756-x

[B20] Cory J. S. Ericsson J. D. (2010). Fungal entomopathogens in a tritrophic context. BioControl 55, 75–88. doi: 10.1007/s10526-009-9247-4

[B21] Cuthbertson A. G. S. Walters K. F. A. (2005). Pathogenicity of the entomopathogenic fungus, *Lecanicillium muscarium*, against the sweet potato whitefly *Bemisia tabaci* under laboratory and glasshouse conditions. Mycopathologia 160, 315–319. doi: 10.1007/s11046-005-0122-2 16244900

[B22] Dao H. T. Beattie G. A. C. Rossman A. Y. Burgess L. W. Holford P. (2016). Four putative entomopathogenic fungi of armoured scale insects on *Citrus* in Australia. Mycol. Prog. 15, 47. doi: 10.1007/s11557-016-1188-6

[B23] Da Silva Santos A. C. Da Silva Lopes R. Goncalves de Oliveira L. Goncalves Diniz A. Shakeel M. de Lina Alves Lima E. A.. (2022). ““Entomopathogenic fungi: current status and prospects,”,” in New and Future Development in Biopesticide Research: Biotechnological Exploration. Eds. Mandal S. D. Ramkumar G. Karthi S. Jin F. (Springer, Sinapore).

[B24] De La Rosa W. Alatorre R. Barrera J. F. Toriello C. (2000). Effect of *Beauveria bassiana* and *Metarhizium anisopliae* (Deuteromycetes) upon the coffee berry borer (Coleoptera: Scolytidae) under field conditions. J. Econ. Entomol. 93, 1409–1414. doi: 10.1603/0022-0493-93.5.1409 11057711

[B25] Dolci P. Guglielmo F. Secchi F. Ozino O. (2006). Persistence and efficacy of *Beauveria brongniartii* strains applied as biocontrol agents against *Melolontha melolontha* in the Valley of Aosta (northwest Italy). J. Appl. Microbiol. 100, 1063–1072. doi: 10.1111/j.1365-2672.2006.02808.x 16630007

[B26] Elbanhawy A. A. Elsherbiny E. A. Abd El-Mageed A. E. Abdel-Fattah G. M. (2019). Potential of fungal metabolites as a biocontrol agent against cotton aphid, *Aphis gossypii* Glover and the possible mechanisms of action. Pest. Biochem. Physiol. 159, 34–40. doi: 10.1016/j.pestbp.2019.05.013 31400782

[B27] Faria M. R. Wraight S. P. (2007). Mycoinsecticides and mycoacaricides: a comprehensive list with worldwide coverage and international classification of formulation types. Biol. Control 43, 237–256. doi: 10.1016/j.biocontrol.2007.08.001

[B28] Gandarilla-Pacheco F. L. Galán-Wong L. J. López-Arroyo J. I. Rodríguez-Guerra R. Quintero-Zapata I. (2013). Optimization of pathogenicity tests for selection of native isolates of entomopathogenic fungi isolated from *Citrus* growing areas of Mexico on adults of *Diaphorina citri* Kuwayama (Hemiptera: Liviidae). Fla. Entomol. 96, 187–195. doi: 10.1653/024.096.0125

[B29] Glare T. R. Caradus J. Gelernter W. Jackson T. Keyhani N. Kohl J.. (2012). Have biopesticides come of age? Trends Biotechnol. 30, 250–258. doi: 10.1016/j.tibtech.2012.01.003 22336383

[B30] Goble T. A. Dames J. F. Hill M. P. Moore S. D. (2010). The effects of farming system, habitat type and bait type on the isolation of entomopathogenic fungi from citrus soils in the eastern Cape Province, South Africa. BioControl 55, 399–412. doi: 10.1007/s10526-009-9259-0

[B31] Goble T. A. Dames J. F. Hill M. P. Moore S. D. (2011). Investigation of native isolates of entomopathogenic fungi for the biological control of three citrus pests. Biocontrol Sci. Tech. 21, 1193–1211. doi: 10.1080/09583157.2011.608907

[B32] Goettel M. S. Koike M. Kim J. J. Aiuchi D. Shinya R. Brodeur J. (2008). Potential of *Lecanicillium* spp. for management of insects, nematodes and plant diseases. J. Invertebr. Pathol. 98, 256–261. doi: 10.1016/j.jip.2008.01.009 18423483

[B33] Gupta A. Nair S. (2020). Dynamics of insect-microbiome interaction influence host and microbial symbiont. Front. Microbiol. 11, 01357. doi: 10.3389/fmicb.2020.01357 PMC7333248 32676060

[B34] Gurung K. Wertheim B. Falcao Salles J. (2019). The microbiome of pest insects: it is not just bacteria. Entomol. Exp. Appl. 167, 156–170. doi: 10.1111/eea.12768

[B35] Hajek A. E. (2007). ““Introduction of a fungus into North America for control of gypsy moth,”,” in Biological Control: A Global Perspective. Eds. Vincent C. Goettel M. S. Lazarovits G. (CAB International, Wallingford, UK), 53–62.

[B36] Hummel R. L. Walgenbach J. F. Barbercheck M. E. Kennedy G. G. Hoyt G. D. Arellano C. (2002). Effects of production practices on soil-borne entomopathogens in western North Carolina vegetable systems. Environ. Entomol. 31, 84–91. doi: 10.1603/0046-225X-31.1.84

[B37] Inglis G. D. Goettel M. S. Butt T. M. Strasser H. (2001). ““Use of hyphomycetous fungi for managing insect pests,”,” in Fungi as Biocontrol Agents – Progress, Problems and Potential. Eds. Butt T. Jackson C. Magan N. (CAB International, Wallingford, UK), 23–69.

[B38] Inyang E. Butt T. M. Ibrahim L. Clarke S. J. Pye B. J. Beckett A.. (1998). The effect of plant growth and topography on the acquisition of conidia of the insect pathogen *Metarhizium anisopliae* by larvae of *Phaedon cochleariae* . Mycol. Res. 102, 1365. doi: 10.1017/S095375629800673X

[B39] Irsad Shahid M. Haq E. Mohamed A. Rizvi P. Q. Kolanthasamy E. (2023). Entomopathogen-based biopesticides: insights into unraveling their potential in insect pest management. Front. Microbiol. 14, 1208237. doi: 10.3389/fmicb.2023.1208237 37564286 PMC10411202

[B40] Jaronski S. T. Jackson M. A. (2012). ““Mass production of entomopathogenic Hypocreales,”,” in Manual of Techniques in Invertebrate Pathology. Ed. Lacey L. A. (Academic Press, San Diego, USA), 257–286.

[B41] Jaronski S. T. Mascarin G. M. (2017). ““Mass production of fungal entomopathogens,”,” in Microbial Control of Insects and Mite Pests. Ed. Lacey L. A. (Academic, Amsterdam), 141–155.

[B42] Kabaluk J. T. Svircev A. M. Goettel M. S. Woo S. G. (2010). The use and regulation of microbial pesticides in representative jurisdictions worldwide (Rome: IOBC Global).

[B43] Keller S. Kessler P. Schweizer C. (2003). Distribution of insect pathogenic soil fungi in Switzerland with special reference to *Beauveria brongniartii* and *Metarhizium anisopliae* . Biocontrol 48, 307–319. doi: 10.1023/A:1023646207455

[B44] Kim J. J. Goettel M. S. Gillespie D. R. (2009). Evaluation of *Lecanicillium longisporum*, Vertalec against the cotton aphid, *Aphis gossypii*, and cucumber powdery mildew, *Sphaerotheca fuliginea* in a greenhouse environment. Crop Protect. 29, 540–544. doi: 10.1016/j.biocontrol.2008.02.003

[B45] Koike M. Shinya R. Aiuchi D. Mori M. Ogino R. Shinomiya H.. (2011). ““Future biological control for soybean cyst nematode,”,” in Soybean Physiology and Biochemistry. Ed. El-Shemy H. A. (Intech, Croatia), 193–208.

[B46] Labbe R. M. Gillespie D. R. Cloutier C. Brodeur J. (2009). Compatibility of an entomopathogenic fungus with a predator and a parasitoid in the biological control of greenhouse whitefly. Biocontrol Sci. Technol. 19, 429–446. doi: 10.1080/09583150902803229

[B47] Lacey L. A. Frutos R. Kaya H. K. Vail P. (2001). Insect pathogens as biological control agents: do they have a future? Biol. Control 21, 230–248. doi: 10.1006/bcon.2001.0938

[B48] Lacey L. A. Grzywacz D. Shapiro-Ilan D. I. Frutos R. Brownbridge M. Goettel M. S. (2015). Insect pathogens as biological control agents: Back to the future. J. Invert. Pathol. 132, 1–41. doi: 10.1016/j.jip.2015.07.009 26225455

[B49] Lacey L. A. Liu T. X. Buchman J. L. Munyaneza J. E. Goolsby J. A. Horton D. R. (2011). Entomopathogenic fungi (Hypocreales) for control of potato psyllid, *Bactericera cockerelli* (Šulc) (Hemiptera: Triozidae) in an area endemic for zebra chip disease of potato. Biol. Control 36, 271–278. doi: 10.1016/j.biocontrol.2010.11.012

[B50] Lomer C. J. Bateman R. P. Dent D. De Groote H. Douro-Kpindou O. K. Kooyman C.. (1999). Development of strategies for the incorporation of biological pesticides into the integrated management of locusts and grasshoppers. Agric. For. Entomol. 1, 71–88. doi: 10.1111/j.1461-9563.1999.tb00001.x

[B51] Lomer C. J. Bateman R. P. Johnson D. L. Langewald J. Thomas M. (2001). Biological control of locusts and grasshoppers. Annu. Rev. Entomol. 46, 667–702. doi: 10.1146/annurev.ento.46.1.667 11112183

[B52] Lopes R. S. Svedese V. M. Portela A. P. A. S. Albuquerque A. C. Luna-Alves Lima E. A. (2011). Virulence and biological aspects of *Isaria javanica* (Frieder & Bally) Samson & Hywell-Jones in *Coptotermes gestroi* (Wasmann) (Isoptera: Rhinotermitidae). Arq. Inst. Biol. 78, 565–572. doi: 10.1590/1808-1657v78p5652011

[B53] Lopez Plantey R. Papura D. Couture C. Thiery D. Bertoldi M. V. Lucero G. S. (2019). Characterization of entomopathogenic fungi from vineyards in Argentina with potential as biological control agents against the European grapevine moth. Lobesia botrana. Biocontrol 64, 501–511. doi: 10.1007/s10526-019-09955-z

[B54] Lu H. L. Wang J. B. Brown M. A. Euerle C. Leger R. J. S. (2015). Identification of *Drosophila* mutants affecting defense to an entomopathogenic fungus. Sci. Rep. 5, 12350. doi: 10.1038/srep12350 26202798 PMC4511952

[B55] Luz C. Bastos Netto M. C. Rocha L. F. N. (2007). *In vitro* susceptibility to fungicides by invertebrate pathogenic and saprobic fungi. Mycopathologia 164, 39–47. doi: 10.1007/s11046-007-9020-0 17574540

[B56] Ma M. Luo J. Li C. Eleftherianos I. Zhang W. Xu L. (2024). A life-and-death struggle: interaction of insects with entomopathogenic fungi across various infection stages. Front. Immunol. 14, 1329843. doi: 10.3389/fimmu.2023.1329843 38259477 PMC10800808

[B57] Maina U. M. Galadima I. B. Gambo F. M. Zakaria D. (2018). A review on the use of entomopathogenic fungi in the management of insect pests of field crops. J. Entomol. Zool. Stud. 6, 27–32.

[B58] Malusa E. Berg G. Biere A. Bohr A. Canfora L. Jungblut A. D.. (2021). A holistic approach for enhancing the efficacy of soil microbial inoculants in agriculture. Glob. J. Agric. Innov. Res. Dev. 8, 176–190. doi: 10.15377/2409-9813.2021.08.14

[B59] Marrone P. G. (2014). ““The market and potential for biopesticides,”,” in Biopesticides: State of the Art and Future Opportunities. Eds. Gross A. D. Coats J. R. Seiber J. N. Duke S. O. (American Chemical Society, Washington), 245–258. 10.1021/jf504252n 25406111

[B60] Marrone P. G. (2019). Pesticidal natural products–status and future potential. Pest Manage. Sci. 75, 2325–2340. doi: 10.1002/ps.5433 30941861

[B61] Meekers E. T. M. Faransen J. J. van Lenteren J. C. (2002). Pathogenicity of *Aschersonia* spp. against whiteflies *Bemisia argentifolii* and *Trialeurodes vaporariorum* . J. Invertebr. Pathol. 81, 1–11. doi: 10.1016/S0022-2011(02)00150-7 12417207

[B62] Meyling N. V. Eilenberg J. (2007). Ecology of the entomopathogenic fungi *Beauveria bassiana* and *Metarhizium anisopliae* in temperate agroecosystems: potential for conservation biocontrol. Biol. Control 43, 145–155. doi: 10.1016/j.biocontrol.2007.07.007

[B63] Milner R. J. Samson P. Morton R. (2003). Persistence of conidia of *Metarhizium anisopliae* in sugarcane fields: effect of isolate and formulation on persistence over 3.5 years. Biocontrol Sci. Technol. 13, 507–516. doi: 10.1080/0958315031000140965

[B64] Moscardi F. Sosa-Gomez D. (2007). ““Microbial control of insect pests of soybean,”,” in Field Manual of Techniques in Invertebrate Pathology: Application and Evaluation of Pathogens for Control of Insects and Other Invertebrate Pests, 2nd ed. Eds. Lacey L. A. Kaya H. K. (Springer, Dordrecht, The Netherlands), 411–426.

[B65] Moussa A. Maixner M. Stephan D. Santoiemma G. Passera A. Mori N.. (2021). Entomopathogenic nematodes and fungi to control *Hyalesthes obsoletus* (Hemiptera: Auchenorrhyncha: Cixiidae). BioControl 66, 523–534. doi: 10.1007/s10526-020-10076-1

[B66] Mustu M. Demirci F. Kaydan M. B. Ülgentürk S. (2015). Laboratory assay of the effectiveness of the entomopathogenic fungus *Isaria farinosa* (Holmsk.) Fries (Sordariomycetes: Hypocreales) against the vine mealybug *Planococcus ficus* (Signoret) (Hemiptera: Pseudococcidae), even under the use of fungicides. Inter. J. Pest Manage. 61, 264–271. doi: 10.1080/09670874.2015.1047811

[B67] Nielsen C. Hajek A. E. (2005). Control of invasive soybean aphid, *Aphis glycines* (Hemiptera: Aphididae), populations by existing natural enemies in New York State, with emphasis on entomopathogenic fungi. Environ. Entomol. 34, 1036–1047. doi: 10.1603/0046-225X(2005)034[1036:COISAA]2.0.CO;2

[B68] Olleka A. Mandour N. Ren S. (2009). Effect of host plant on susceptibility of whitefly *Bemisia tabaci* (Homoptera: Aleyrodidae) to the entomopathogenic fungus *Beauveria bassiana* (Ascomycota: Hypocreales). Biocontrol Sci. Technol. 19, 717–727. doi: 10.1080/09583150903042843

[B69] Ortiz-Urquiza A. Keyhani N. O. (2013). Action on the surface: entomopathogenic fungi versus the insect cuticle. Insects 4, 357–374. doi: 10.3390/insects4030357 26462424 PMC4553469

[B70] Ownley B. H. Griffin M. R. Klingeman W. E. Gwinn K. D. Moulton J. K. Pereira R. M. (2008). *Beauveria bassiana*: endophytic colonization and plant disease control. J. Invertebr. Pathol. 98, 267–270. doi: 10.1016/j.jip.2008.01.010 18442830

[B71] Ownley B. H. Gwinn K. D. Vega F. E. (2010). Endophytic fungal entomopathogens with activity against plant pathogens: ecology and evolution. Biocontrol 55, 113–128. doi: 10.1007/s10526-009-9241-x

[B72] Ownley B. H. Pereira R. M. Klingeman W. E. Quigley N. B. Leckie B. M. (2004). ““*Beauveria bassiana*, a dual purpose biocontrol organism with activity against insect pests and plant pathogens,”,” in Emerging Concepts in Plant Health Management. Eds. Lartey R. T. Caesar A. J. (Research Signpost, Kerala, India), 255–269.

[B73] Pal S. Leger R. J. S. Wu L. P. (2007). Fungal peptide Destruxin a plays a specific role in suppressing the innate immune response in *Drosophila melanogaster* . J. Biol. Chem. 282, 8969–8977. doi: 10.1074/jbc.M605927200 17227774

[B74] Pell J. K. (2007). ““Ecological approaches to pest management using entomopathogenic fungi: concepts, theory, practice and opportunities,”,” in Use of Entomopathogenic Fungi in Biological Pest Management. Eds. Ekesi S. Maniania N. K. (Research Signpost, Kerala, India), 145–177.

[B75] Pell J. K. Hannam J. J. Steinkraus D. C. (2010). Conservation biological control using fungal entomopathogens. Biocontrol 55, 187–198. doi: 10.1007/s10526-009-9245-6

[B76] Pereault R. J. Whalon M. E. Alston D. G. (2009). Field efficacy of entomopathogenic fungi and nematodes targeting caged last-instar plum curculio (Coleoptera: Curculionidae) in Michigan cherry and apple orchards. Environ. Entomol. 38, 1126–1134. doi: 10.1603/022.038.0420 19689891

[B77] Qu S. Wang S. (2018). Interaction of entomopathogenic fungi with the host immune system. Dev. Comp. Immunol. 83, 96–103. doi: 10.1016/j.dci.2018.01.010 29355579

[B78] Quesada-Moraga E. Navas-Cortez J. A. Maranhao E. A. Ortiz-Urquiza A. Santiago Alvarez C. (2007). Factors affecting the occurrence and distribution of entomopathogenic fungi in natural and cultivated soils. Mycol. Res. 111, 947–966. doi: 10.1016/j.mycres.2007.06.006 17766099

[B79] Quesada-Moraga E. Munoz-Ledesma F. J. Santiago-Alvarez C. (2009). Systemic protection of *Papaver somniferum* L. against Iraella luteipes (Hymenoptera: Cynipidae) by an endophytic strain of *Beauveria bassiana* (Ascomycota: Hypocreales). Environ. Entomol 38, 723–730. doi: 10.1603/022.038.0324 19508781

[B80] Rocha L. F. N. Luz C. (2009). Utility of six fungicides for selective isolation of *Evlachovaea* spp. and *Tolypocladium cylindrosporum* . Mycopathologia 167, 341–350. doi: 10.1007/s11046-009-9186-8 19205922

[B81] Rondot Y. Reineke A. (2018). Endophytic *Beauveria bassiana* in grapevine *Vitis vinifera* (L.) reduces infestation with piercing-sucking insects. Biol. Control 116, 82–89. doi: 10.1016/j.biocontrol.2016.10.006

[B82] Roy H. E. Brodie E. L. Chandler D. Goettel M. Pell J. Wajnberg E.. (2010a). Hidden depths: understanding the evolution and ecology of fungal entomopathogens. Biocontrol 55, 1–6. doi: 10.1007/s10526-009-9244-7

[B83] Roy H. E. Vega F. E. Chandler D. Goettel M. S. Pell J. K. Wajnberg E. (2010b). The Ecology of Fungal Entomopathogens (Dordrecht, The Netherlands: Springer). doi: 10.1007/978-90-481-3966-8

[B84] Santoro P. H. Neves P. M. O. J. Silva R. Z. Akimi S. Janaína Zorzetti J. (2005). *Beauveria bassiana* (Bals.) Vuill. Spores production in biphasic process utilizing different liquid media. Semina Cienc. Agrar. 26, 313–320. doi: 10.5433/1679-0359.2005v26n3p313

[B85] Sayed S. El-Shehawi A. Al-Otaibi S. El-Shazly S. Al-Otaibi S. Ibrahim R.. (2020). Isolation and efficacy of the endophytic fungus, *Beauveria bassiana* (Bals.) Vuillemin on grapevine aphid, *Aphis illinoisensis* Shimer (Hemiptera: Aphididae) under laboratory conditions. Egypt J. Biol. Pest Control 30, 38. doi: 10.1186/s41938-020-00234-z

[B86] Shapiro-Ilan D. I. Gardner W. Fuxa J. R. Wood B. W. Nguyen K. Adams B. J.. (2003). Survey of entomopathogenic nematodes and fungi endemic to pecan orchards of the southeastern US and their virulence to the pecan weevil (Coleoptera: Curculionidae). Environ. Entomol. 32, 187–195. doi: 10.1603/0046-225X-32.1.187

[B87] Shapiro-Ilan D. I. Jackson M. Reilly C. C. Hotchkiss M. W. (2004). Effects of combining an entomopathogenic fungi or bacterium with entomopathogenic nematodes on mortality of *Curculio caryae* (Coleoptera: Curculionidae). Biol. Cont. 30, 119–126. doi: 10.1016/j.biocontrol.2003.09.014

[B88] Sharma L. Goncalves F. Oliveira I. Torres L. Marques G. (2018). Insect-associated fungi from naturally mycosed vine mealybug *Planococcus ficus* (Signoret) (Hemiptera: Pseudococcidae). Biocontrol Sci. Technol. 28, 122–141. doi: 10.1080/09583157.2018.1428733

[B89] Singh D. Kour Raina T. Singh J. (2017). Entomopathogenic fungi: An effective biocontrol agent for management of insect populations naturally. J. Pharm. Sci. Res. 9, 830–839.

[B90] Spescha A. Weibel J. Wyser L. Brunner M. Hess Hermida M. Moix A.. (2023a). Combining entomopathogenic Pseudomonas bacteria, nematodes and fungi for biological control of a below-ground insect pest. Agric. Eco. Environ. 348, 108414. doi: 10.1016/j.agee.2023.108414

[B91] Spescha A. Zwyssig M. Hess Hermida M. Moix M. Bruno P. Enkerli J.. (2023b). When competitors join forces: consortia of entomopathogenic microorganisms increase killing speed and mortality in leaf- and root-feeding insect hosts. Microb. Ecol. 86, 1947–1960. doi: 10.1007/s00248-023-02191-0 36849610 PMC10497674

[B92] Steinkraus D. C. (2007a). ““Management of aphid populations in cotton through conservation: delaying insecticide spraying has its benefits,”,” in Biological Control: A Global Perspective. Eds. Vincent C. Goettel M. S. Lazarovits G. (CAB International, Wallingford, UK), 383–391.

[B93] Steinkraus D. C. (2007b). ““Documentation of naturally occurring pathogens and their impact in agroecosystems,”,” in Field Manual of Techniques in Invertebrate Pathology: Application and Evaluation of Pathogens for Control of Insects and Other Invertebrate Pests, 2nd ed. Eds. Lacey L. A. Kaya H. K. (Springer, Dordrecht, The Netherlands), 267–281.

[B94] Thakre M. Thakur M. Malik N. Ganger S. (2011). Mass scale cultivation of entomopathogenic fungus *Nomuraea rileyi* using agricultural products and agro wastes. J. Biopest. 4, 176–179. doi: 10.57182/jbiopestic

[B95] Thomas M. B. (2000). ““Development of a mycoinsecticide for biological control of locusts in Southern Africa,”,” in Research Priorities for Migrant Pests of Agriculture in Southern Africa. Proceedings of a DFID/NRI/ARC-PPRI Workshop. Eds. Cheke R. A. Rosenberg L. J. Kieser M. E. (Natural Resources Institute, Chatham, UK), 173–182.

[B96] Townsend R. J. Nelson T. L. Jackson T. A. (2010). *Beauveria brongniartii* – a potential biocontrol agent for use against manuka beetle larvae damaging dairy pastures on Cape Foulwind. N. Z. Plant Protect. 63, 224–228. doi: 10.30843/nzpp.2010.63

[B97] Townsend R. J. O’Callaghan M. Johnson V. W. Jackson T. A. (2003). Compatibility of microbial control agents *Serratia entomophila* and *Beauveria bassiana* with selected fertilisers. N. Z. Plant Protect. 56, 118–122. doi: 10.30843/nzpp.2003.56

[B98] Ugine T. A. Wraight S. P. Sanderson J. P. (2007a). Effects of manipulating spray application parameters on efficacy of the entomopathogenic fungus *Beauveria bassiana* against western flower thrips, Frankliniella occidentalis, infesting greenhouse impatiens crops. Biocontrol Sci. Technol. 17, 193–219. doi: 10.1080/09583150600937618

[B99] Ugine T. A. Wraight S. P. Sanderson J. P. (2007b). A tritrophic effect of host plant on susceptibility of western flower thrips to the entomopathogenic fungus *Beauveria bassiana* . J. Invertebr. Pathol. 96, 162–172. doi: 10.1016/j.jip.2007.05.004 17572438

[B100] Vega F. E. Meyling N. V. Luangsa-ard J. J. Blackwell M. (2012). ““Fungal entomopathogens,”,” in Insect Pathology, 2nd ed. Eds. Vega F. E. Kaya H. K. (Academic Press, San Diego), 172–220.

[B101] Wang C. Leger R. J. S. (2006). A collagenous protective coat enables *Metarhizium anisopliae* to evade insect immune responses. Proc. Natl. Acad. Sci. 103, 6647–6652. doi: 10.1073/pnas.0601951103 16614065 PMC1458935

[B102] Wang H. Peng H. Li W. Cheng P. Gong M. (2021). The toxins of *Beauveria bassiana* and the strategies to improve their virulence to insects. Front. Microbiol. 12, 705343. doi: 10.3389/fmicb.2021.705343 34512581 PMC8430825

[B103] Wei G. Lai Y. Wang G. Chen H. Li F. Wang S. (2017). Insect pathogenic fungus interacts with the gut microbiota to accelerate mosquito mortality. Proc. Natl. Acad. Sci. U.S.A. 114, 5994–5999. doi: 10.1073/pnas.1703546114 28533370 PMC5468619

[B104] Wekesa V. W. Maniania N. K. Knapp M. Boga H. I. (2005). Pathogenicity of *Beauveria bassiana* and *Metarhizium anisopliae* to the tobacco spider mite *Tetranychus evansi* . Exp. Appl. Acarol. 36, 41–50. doi: 10.1007/s10493-005-0508-3 16082922

[B105] Wraight S. P. Inglis G. D. Goettel M. S. (2007a). ““Fungi,”,” in Field Manual of Techniques in Invertebrate Pathology: Application and Evaluation of Pathogens for Control of Insects and Other Invertebrate Pests, 2nd ed. Eds. Lacey L. A. Kaya H. K. (Springer, Dordrecht, The Netherlands), 223.

[B106] Wraight S. P. Ramos M. E. (2002). Application parameters affecting field efficacy of *Beauveria bassiana* foliar treatments against Colorado potato beetle *Leptinotarsa decemlineata* . Biol. Control 23, 164–178. doi: 10.1006/bcon.2001.1004

[B107] Wraight S. P. Sporleder M. Poprawski T. J. Lacey L. A. (2007b). ““Application and evaluation of entomopathogens in potato,”,” in Field Manual of Techniques in Invertebrate Pathology: Application and Evaluation of Pathogens for Control of Insects and Other Invertebrate Pests, 2nd ed. Eds. Lacey L. A. Kaya H. K. (Springer, Dordrecht, The Netherlands), 329–359.

[B108] Xu J. Xu X. Shakeel M. Li S. Wang S. Zhou X.. (2017). The entomopathogenic fungi *Isaria fumosorosea* plays a vital role in suppressing the immune system of *Plutella xylostella*: RNA-Seq and DGE analysis of immunity-related genes. Front. Microbiol. 8, 1421. doi: 10.3389/fmicb.2017.01421 28804478 PMC5532397

[B109] Zimmermann G. (1992). ““Use of the fungus, *Beauveria brongniartii*, for the control of European cockchafers, Melolontha spp. in Europe,”,” in Use of Pathogens in Scarab Pest Management. Eds. Jackson T. A. Glare T. R. (Intercept Limited, Hampshire, UK), 199–208.

[B110] Zimmermann G. (2008). The entomopathogenic fungi *Isaria farinosa* (formerly *Paecilomyces farinosus*) and the *Isaria fumosorosea* species complex (formerly known as *Paecilomyces fumosoroseus*): biology, ecology and its use in biological control. Biocontrol Sci. Technol. 18, 865–901. doi: 10.1080/09583150802471812

